# ENTH and ANTH domain proteins participate in AP2-independent clathrin-mediated endocytosis

**DOI:** 10.1242/jcs.167726

**Published:** 2015-06-01

**Authors:** Paul T. Manna, Catarina Gadelha, Amy E. Puttick, Mark C. Field

**Affiliations:** 1 Department of Pathology, University of Cambridge, Tennis Court Road, Cambridge CB2 1QP, UK; 2 School of Life Sciences, University of Nottingham, Queen's Medical Centre, Nottingham NG7 2UH, UK; 3 Division of Biological Chemistry and Drug Discovery, University of Dundee, Dundee DD1 5EH, UK

**Keywords:** Endocytosis, Evolution, Phosphoinositide, Trafficking, Trypanosome

## Abstract

Clathrin-mediated endocytosis (CME) is a major route of entry into eukaryotic cells. A core of evolutionarily ancient genes encodes many components of this system but much of our mechanistic understanding of CME is derived from a phylogenetically narrow sampling of a few model organisms. In the parasite *Trypanosoma brucei*, which is distantly related to the better characterised animals and fungi, exceptionally fast endocytic turnover aids its evasion of the host immune system. Although clathrin is absolutely essential for this process, the adaptor protein complex 2 (AP2) has been secondarily lost, suggesting mechanistic divergence. Here, we characterise two phosphoinositide-binding monomeric clathrin adaptors, *T. brucei* (Tb)EpsinR and TbCALM, which in trypanosomes are represented by single genes, unlike the expansions present in animals and fungi. Depletion of these gene products reveals essential, but partially redundant, activities in CME. Ultrastructural analysis of TbCALM and TbEpsinR double-knockdown cells demonstrated severe defects to clathrin-coated pit formation and morphology associated with a dramatic inhibition of endocytosis. Depletion of TbCALM alone, however, produced a distinct lysosomal segregation phenotype, indicating an additional non-redundant role for this protein. Therefore, TbEpsinR and TbCALM represent ancient phosphoinositide-binding proteins with distinct and vital roles in AP2-independent endocytosis.

## INTRODUCTION

Endocytosis is an essential cellular process in eukaryotes. In animal cells, multiple endocytic pathways exist, but the best understood requires the vesicle coat protein clathrin. Intensive study in both animal and yeast systems has identified many, if not most, of the proteins involved in clathrin-mediated endocytosis (CME) (e.g. Borner et al., 2006; Borner et al., 2012). CME is evolutionarily ancient, with components of this pathway found throughout the eukaryotes, and most notably clathrin itself, with the exception of the extremely reduced genomes of microsporidia (Barlow et al., 2014), is present in all eukaryotic genomes so far examined. Recruitment of clathrin to the plasma membrane, concentration of endocytic cargo and clathrin-coated pit (CCP) formation all depend on an array of accessory proteins or adaptors. The heterotetrameric adaptor protein complex 2 (AP2) binds clathrin and functions as a central adaptor hub in CME, mediating membrane recruitment through phosphatidylinositol 4,5-bisphosphate [PtdIns(4,5)*P*_2_] binding, and cargo selection by recognition of endocytic motifs on cargo proteins (e.g. [D/E]xxxL[LI] or Yxxɸ, where ɸ denotes a large hydrophobic residue) (Lafer, 2002; Schmid et al., 2006; Jackson et al., 2010; McMahon and Boucrot, 2011). In animal cells, depletion of the AP2 complex is sufficient to block CCP formation, suggesting that CME is indeed dependent on AP2 (Boucrot et al., 2010).

Outside metazoa, the AP2 complex is consistently found to localise with clathrin at the plasma membrane (Elde et al., 2005), but its requirement in CME is less clear. Early studies in yeast found no significant endocytic defect upon depletion of the AP2 complex (Yeung et al., 1999; Huang et al., 1999), suggesting that there was no absolute requirement for AP2 in CME. More recently, a potential role for the AP2 complex as a cargo-specific adaptor was identified in a yeast genetic screen for resistance to an endocytosed toxin (Carroll et al., 2009). However, the scale of involvement of AP2 in yeast CME appears to be highly reduced compared to animals. Similarly, studies in *Dictyostelium* find association of AP2 with clathrin structures at the plasma membrane (Sosa et al., 2012; Macro et al., 2012) but little effect of AP2 depletion on CME (Macro et al., 2012).

In addition to the heterotetrameric adaptor protein complexes, numerous monomeric clathrin adaptors are known. Among these the most widely conserved are phosphatidylinositol phosphate (PtdInsP)-binding proteins containing epsin N-terminal homology (ENTH) and AP180 N-terminal homology (ANTH) domains. The ENTH domain is present in metazoan epsins (Eps15-interacting) and EpsinR (epsin-related) proteins (CLINT1 in humans) along with their yeast counterparts ENT1–ENT5. The ANTH domain is found in the clathrin assembly lymphoid myeloid leukaemia protein (CALM, also known as PICALM) and its neuronal-specific homologue, adaptor protein of 180 kDa (AP180, also known as SNAP91), as well as in huntingtin-interacting protein 1 (HIP1) and HIP-related (HIP1R), and their yeast counterparts YAP180-1 and YAP180-2 and SLA2. In yeast and animals, the epsins function in CME, whereas epsinR localises primarily to the Golgi complex and mediates trafficking between endosomes and the trans-Golgi network (TGN) (Kalthoff et al., 2002; Mills et al., 2003; Hirst et al., 2003). Outside of the opisthokonts, EpsinR is the sole representative of this protein family and in *Trypanosoma brucei* plays a role in endocytosis from the plasma membrane (Gabernet-Castello et al., 2009). Likewise, CALM and AP180 represent the ancestral ANTH domain proteins, with a single homologue present in most eukaryotes (De Craene et al., 2012). AP180 has the ability to recruit clathrin to lipid bilayers *in vitro* (Ford et al., 2001) but again homologues are dispensable for CME in many organisms. In yeast, simultaneous depletion of EPN1 and EPN2, and both the AP180 or CALM homologues (YAP180-1 and YAP180-2) is sufficient to block CME, suggesting a potential mechanism for AP2-independent CME involving redundant functions of ENTH and ANTH domain proteins (Maldonado-Báez et al., 2008).

*Trypanosoma brucei*, the causative agent of sleeping sickness and nagana, is unusual in its ability to thrive within the mammalian bloodstream, successfully evading the host immune response. Key to this success is the dense protective surface coat of glycosylphosphatidylinositol (GPI)-anchored variant surface glycoprotein (VSG). Regular antigenic variation of the VSG coat is coupled to extremely rapid endocytosis and removal of bound host antibodies, allowing the parasite to reach extremely high levels of parasitaemia (Barry and McCulloch, 2001; Engstler et al., 2007; Manna et al., 2014). The high endocytic flux of bloodstream form *T. brucei* depends entirely upon CME, which is the sole mechanism of entry into the endomembrane system and is essential to parasite viability (Allen et al., 2003). Although *T. brucei* has a generally conventional endomembrane system, the molecular mechanisms underlying the earliest events in CCP formation appear highly divergent, and in particular *T. brucei* and its close relatives have dispensed entirely with the AP2 complex (Berriman et al., 2005; Field et al., 2007; Manna et al., 2013). A recent proteomic survey of clathrin-interacting proteins identified a cohort of trypanosomatid-specific proteins, suggesting dynamic evolution of this system (Adung'a et al., 2013), although there is clear maintenance of several conserved features, such as the involvement of EpsinR (Gabernet-Castello et al., 2009). In addition to EpsinR (Tb927.11.670), the trypanosome genome encodes a single ANTH domain protein, *T. brucei* (Tb)CALM (Tb927.11.4800), which also retains features suggestive of a role in CME. Here, we examine the contributions of TbEpsinR and TbCALM to endocytic activity in *T. brucei* and, by extension, further characterise the clearest example of wholly AP2-independent clathrin-mediated endocytosis.

## RESULTS

### A reduced clathrin adaptor gene cohort in *T. brucei*

**Fig. 1.**
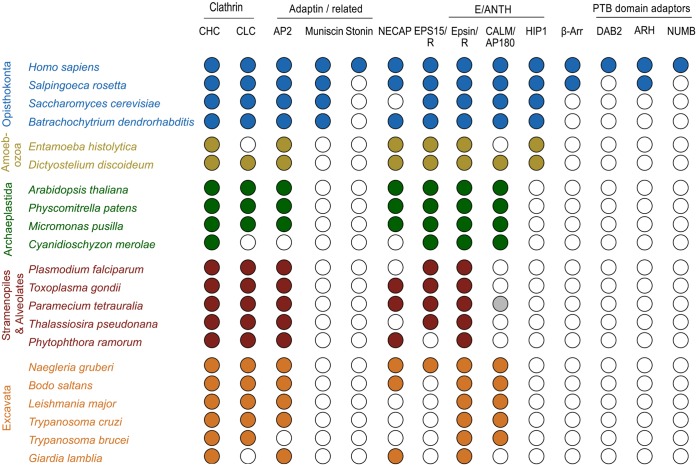
**Phylogenetic distribution of early acting CME partners.** Coulson plot demonstrating presence or absence of genes encoding early acting clathrin-associated proteins across a range of eukaryotes. Filled circles indicate genes identified with high confidence, open circles indicate genes not found, the grey circle indicates a gene identified with low confidence. Rows are taxa and columns are predicted proteins. Supergroups are coloured for clarity. A conserved core machinery is apparent, with an exceptional level of gene loss in *T. brucei*. Plot generated using Coulson Plot Generator (Field et al., 2013). CHC, clathrin heavy chain; CLC, clathrin light chain; β-arr, arrestin β.

Previous studies have provided a good understanding of the early emergence of CME and the broad conservation of many gene families involved. From these studies it emerged that *T. brucei* shows an unusual frequency of secondary loss of CME genes, likely the result of selective pressure for very rapid endocytosis (Field et al., 2007). Owing to recent advances in our understanding of the early stages of clathrin-coated vesicle formation and increased availability of eukaryotic genomes, we revisited and extended these earlier analyses, focussing on genes involved in clathrin recruitment and cargo selection (Fig. 1; supplementary material Table S1).

As previously reported, the *T. brucei* genome lacks all AP2 complex subunits, as well as proposed endocytic initiators EPS15 and EPS15R (also known as EPS15L1 in mammals) and muniscin family proteins (Field et al., 2007; Manna et al., 2013). The loss of Eps15 in particular occurred at the base of the kinetoplastids and suggests divergent mechanisms for endocytic pit initiation throughout this clade. The precise role of the muniscins in CCP formation remains a matter of debate; however, it is worth noting that true muniscins, that is proteins having both a mu homology and a BAR domain, are restricted to the opisthokonts (animals and fungi). Recently, the muniscin family has been shown to be the opisthokont-specific remnant of an ancient pan-eukaryotic adaptor-related protein complex named TSET, for which a detailed phylogenetic analysis has been reported (Hirst et al., 2014). Our analysis also highlights for the first time a further pan-eukaryotic CME component in the NECAP gene family. NECAP is also lost from the trypanosomatids, an event predating the loss of the AP2 complex from salivarian trypanosomes, hinting at potential relaxation or alterations of AP2 function preceding its loss. Importantly, this analysis supports previous suggestions that *T. brucei* has an unusually reduced cohort of endocytic clathrin adaptor and accessory genes, being restricted to one CALM and one EpsinR homologue (Field et al., 2007).

### TbCALM sequence conservation and endocytic localisation

**Fig. 2.**
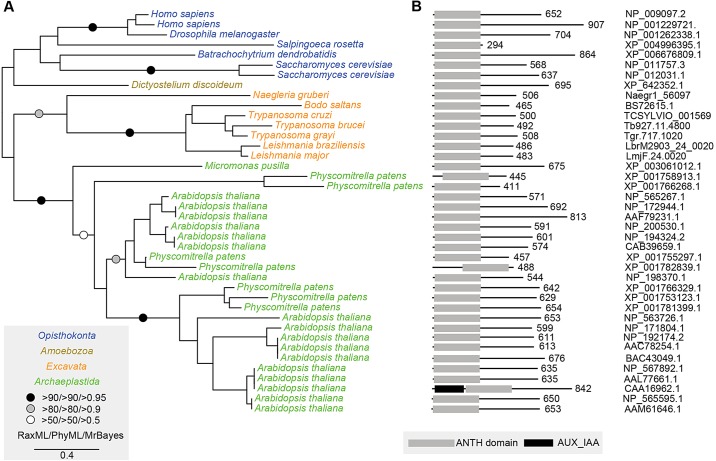
**Phylogenetic analysis and domain structure of CALM proteins.** (A) Phylogenetic tree of predicted protein sequences for CALM orthologues across eukaryotes. Supergroups are coloured for clarity. The topology shown is the best scoring maximum likelihood topology (RaxML). The box provides a key to symbols to indicate statistical support. (B) Domain architecture of CALM proteins corresponding to taxa in A. Grey boxes show the position of the ANTH domain; the protein length in amino acids is stated on the right. A sequence identified from *P. tetraurelia* could not be reliably resolved and was therefore excluded. A high degree of conservation is seen in general domain architecture, although excavates encode CALM proteins that are slightly shorter than average.

Although the trypanosome EpsinR homologue has been studied in some detail (Gabernet-Castello et al., 2009), the trypanosome CALM homologue TbCALM is uncharacterised. To assess the conservation of TbCALM, we gathered CALM sequences from across the eukaryotes for comparison. CALM sequences were identified in all of the major eukaryotic supergroups (Fig. 1) although the only representative identified from the chromalveolates was a low significance match in *Paramecium tetraurelia*, with apparent loss of this gene family from other members of this supergroup, including the closely related *Tetrahymena thermophila* (data not shown). Phylogenetic reconstruction of CALM evolution using sequences from representative taxa revealed a number of lineage specific duplications and expansions, for example in *S. cerevisiae*, *A. thaliana* and *P. patens*, with no evidence for lateral gene transfer (Fig. 2A). Despite expansions in CALM gene number, identified sequences showed a high degree of conservation in domain architecture and length (Fig. 2B). Although it is slightly shorter than the majority of CALM sequences, the identified CALM homologues from the Excavata, the supergroup to which trypanosomes belong, do not appear to have additional unusual features (Fig. 2B). Indeed, there has been surprisingly little apparent acquisition of accessory domains throughout CALM evolution. Overall, TbCALM is a well-conserved member of the CALM gene family, suggesting conserved function with characterised CALM proteins.

**Fig. 3.**
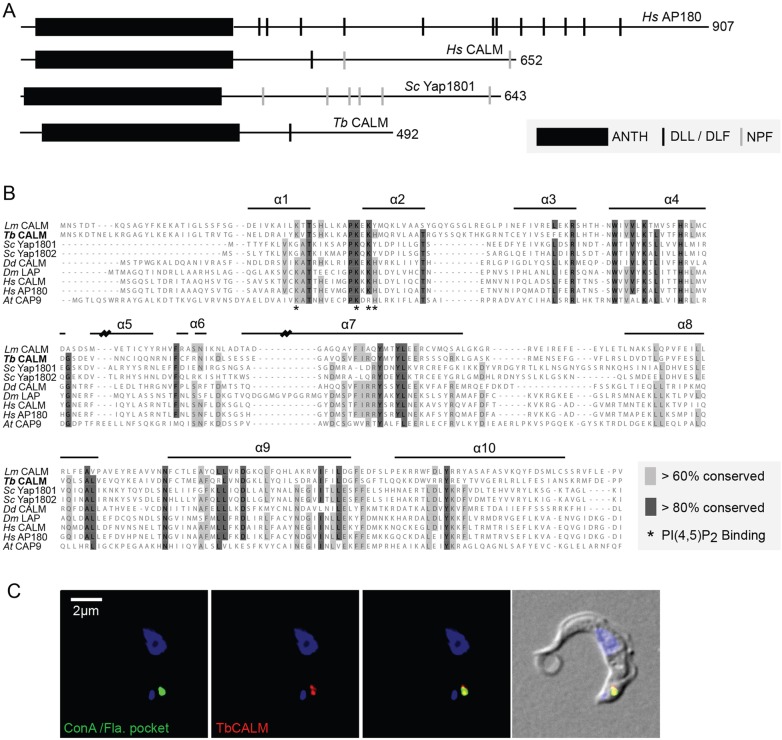
**Conservation of key sequence features and endocytic localisation in TbCALM.** (A) Overview of CALM and AP180 proteins from human (*Hs*), yeast (*Saccharomyces cerevisiae*, *Sc*) and *T. brucei*. The extent of the ANTH domain is indicated (black box) along with the distribution of putative binding sites for clathrin and adaptors (black and grey bars). Clathrin and adaptor sites are well conserved along with general domain architecture. (B) Multiple sequence alignment of CALM proteins from across eukaryotes. Residues highlighted in dark grey are conserved in over 80% of the included sequences, light grey indicates greater than 60% conservation. Taxa are indicated to the left and horizontal bars above the sequence indicate the positions of secondary structural features (Ford et al., 2001). Residues identified as important for PtdIns(4,5)*P*_2_ binding are denoted with asterisks below the sequence. PtdIns(4,5)*P*_2_-binding residues are well conserved. (C) Immunofluorescence localisation of endogenous-locus-tagged TbCALM–GFP in bloodstream form *T. brucei*. Incubation with ConA at 4°C specifically labels the flagellar pocket (green); anti-GFP staining (red) shows TbCALM–GFP colocalised with the flagellar pocket, consistent with an endocytic function. DNA is stained with DAPI to show the nucleus and kinetoplast (blue).

Finally, we focused on the TbCALM sequence in relation to well-characterised homologues. TbCALM retains the conserved domain architecture, consisting of an N-terminal ANTH domain and a predicted disordered C-terminal domain (Fig. 3A). Within the ANTH domain a PtdIns(4,5)*P*_2_-binding motif is located in the loop joining helices 1 and 2. Sequence alignment of TbCALM against several representative homologues demonstrates conservation of key residues within the PtdIns(4,5)*P*_2_-binding motif (Fig. 3B), suggesting conserved PtdIns(4,5)*P*_2_-binding activity for TbCALM. Furthermore, a trypanosome clathrin-binding motif, earlier identified in the clathrin-binding TbEpsinR (Gabernet-Castello et al., 2009) is also found within the C-terminal disordered region of TbCALM, suggesting a role in CME.

For localisation of TbCALM, the gene was tagged at its endogenous locus with either three HA epitopes in tandem or GFP (Fig. 3; supplementary material Fig. S1). Both tags indicated that TbCALM is restricted to the flagellar pocket region of the cell. The flagellar pocket is a functional specialization of the trypanosome surface membrane at the base of its flagellum, where all endocytosis and exocytosis take place. Flagellar pocket localization was demonstrated by colocalisation with a fluorescently labelled lectin, concanavalin A (ConA), a widely used marker for endocytosis in *T. brucei*, which accumulates in the flagellar pocket lumen and membrane when incubated with cells at 4°C (Fig. 3C). The location of TbCALM differs from the distribution of TbEpsinR, which resides more extensively throughout the endocytic system, although some of the protein is associated with clathrin-coated structures at and near the pocket (Gabernet-Castello et al., 2009).

### PtdInsP-dependant membrane targeting of TbCALM and TbEpsinR

Whilst the phosphoinositide composition of the bloodstream form trypanosome flagellar pocket is unknown, a recent report demonstrated enrichment of PtdIns(4,5)*P*_2_ at the flagellar pocket of the insect stage parasite (Demmel et al., 2014). TbEpsinR and TbCALM show conservation of residues important for phosphoinositide binding, suggesting similar membrane targeting mechanisms to their opisthokont orthologues (Fig. 2; Gabernet-Castello et al., 2009).

**Fig. 4.**
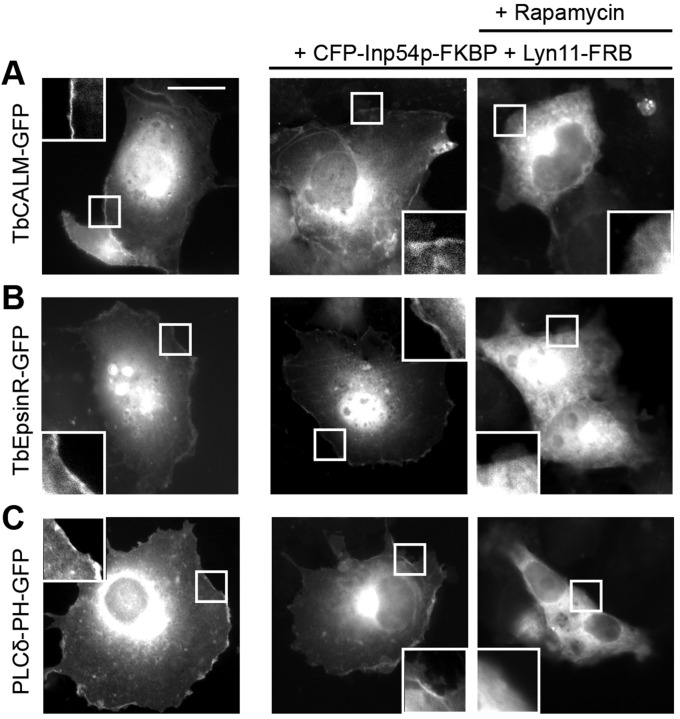
**PtdIns(4,5)*P*_2_ dependence of TbEpsinR and TbCALM membrane targeting.** COS-7 cells grown on glass coverslips were transfected with (A) TbCALM–GFP, (B) TbEpsinR–GFP or (C) PLCδ-PH–GFP alone (left panels) or together with a bipartite, rapamycin inducible, PtdIns(4,5)*P*_2_ depletion system (centre and right panels). Addition of rapamycin (10 μM) (right panels) leads to specific depletion of plasma membrane PtdIns(4,5)*P*_2_, confirmed by the dissociation of PLCδ-PH–GFP from the plasma membrane (C). Insets are magnified images of the boxed regions encompassing plasma membrane. Scale bar: 10 μm. Both TbEpsinR and TbCALM show rapamycin-induced membrane dissociation consistent with some PtdIns(4,5)*P*_2_-binding activity.

To assess the potential phosphoinositide-dependent membrane targeting of trypanosome ENTH and ANTH domain proteins, mammalian expression constructs were made encoding TbCALM or TbEpsinR with C-terminal GFP fusions. TbCALM–GFP expressed in COS-7 cells gave a largely perinuclear distribution with some clear plasma membrane association (Fig. 4). TbEpsinR–GFP displayed more pronounced plasma membrane association, together with small cytoplasmic punctae (Fig. 4) colocalising with clathrin heavy chain, suggesting a location at clathrin-coated vesicles (supplementary material Fig. S2). The plasma membrane localisation of both proteins is suggestive of an interaction with PtdIns(4,5)*P*_2_ in line with the conserved PtdIns(4,5)*P*_2_-binding site seen in TbCALM, but is somewhat unexpected for TbEpsinR, because its mammalian homologue shows selectivity for PtdIns(4)*P* and a more perinuclear distribution (Kalthoff et al., 2002; Mills et al., 2003; Hirst et al., 2003).

To directly address the role of PtdIns(4,5)*P*_2_ in the plasma membrane recruitment of both TbCALM–GFP and TbEpsinR–GFP, we employed a rapamycin-inducible dimerisation system, leading to membrane recruitment of an inositol polyphosphate 5-phosphatase (Inp45p), which selectively cleaves the phosphate at the 5 position of plasma membrane PtdIns(4,5)*P*_2_ following rapamycin addition (Suh et al., 2006). As a control, we also expressed the pleckstrin homology (PH) domain from phospholipase Cδ fused to GFP (PLC_δ_-PH–GFP). As expected the PLC_δ_-PH–GFP construct showed plasma membrane localisation in the absence of rapamycin (Fig. 4C). When co-expressed with the membrane targeting Lyn11–FRB construct and CFP–Inp45p-FKBP, PLC_δ_-PH–GFP dissociated from the membrane following rapamycin treatment and PtdIns(4,5)*P*_2_ depletion (Fig. 4C). Similarly, rapamycin treatment led to a loss of both TbCALM–GFP and TbEpsinR–GFP from the plasma membrane (Fig. 4AB), supporting a specific role for PtdIns(4,5)*P*_2_ in their membrane recruitment. It is, however, likely that additional factors control the distribution of these two proteins in *T. brucei* as their localisations are distinct (Fig. 3 and Gabernet-Castello et al., 2009).

### TbCALM and TbEpsinR are essential in *T. brucei*

**Fig. 5.**
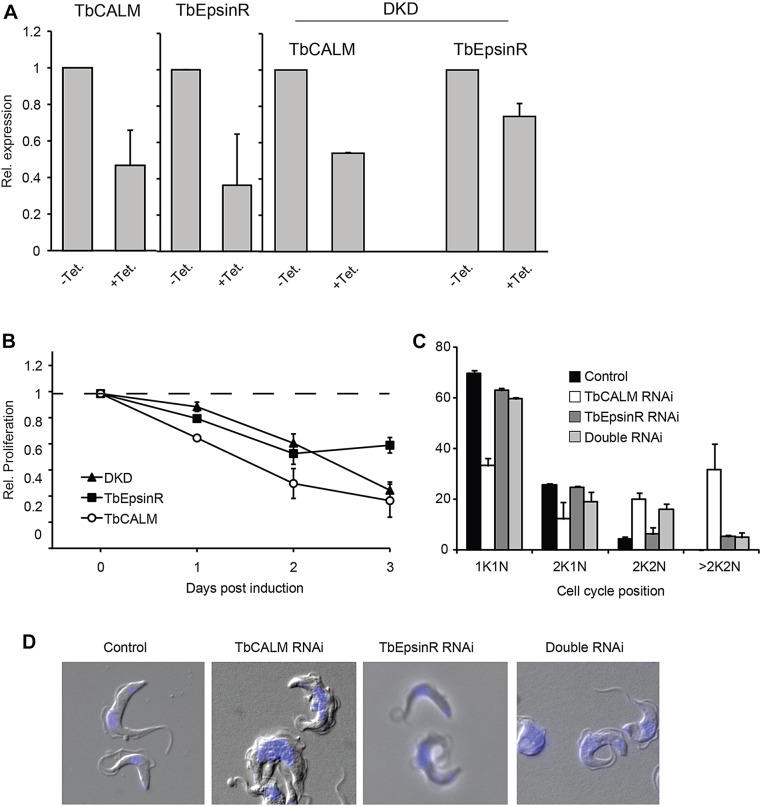
**Proliferative and morphological defects following TbCALM and TbEpsinR depletion.** (A) qRT-PCR analysis of TbCALM and TbEpsinR mRNA expression following 48 h of RNAi induction (+Tet.) in single marker bloodstream-form cells harbouring RNAi constructs targeting either TbCALM, TbEpsinR or both TbCALM and TbEpinR together (DKD). mRNA levels, normalised to β-tubulin, are expressed as relative expression compared to non-induced cells. Data are expressed as mean±s.e.m. from three independent inductions. (B) Proliferation rate of RNAi-induced cells normalised to non-induced cells. Data are mean±s.e.m. from three independent inductions. (C) Cell cycle analysis following 48 h of RNAi induction. xK, number of kinetoplasts; yN, number of nuclei. Control is a parental cell line, shown as black bars; TbCALM RNAi is shown as white bars; TbEpsinR RNAi is dark grey bars; double RNAi is light-grey bars. Data are mean±s.d. from two independent inductions with at least 100 cells counted per specimen per induction. (D) Morphology following depletion of TbCALM and/or TbEpsinR. DIC images of cells after 48 h of RNAi induction; DNA is stained with DAPI (blue).

We next generated transgenic cell lines in which RNA interference (RNAi)-mediated depletion of TbCALM and TbEpsinR (individually or together) could be induced by the addition of tetracycline. Induction of RNAi in these cells causes a reduction in mRNA levels [assessed by quantitative real-time PCR (qRT-PCR); Fig. 5A] and an inhibition of proliferation (Fig. 5B). The proliferative defect caused by silencing of TbCALM and/or *TbEpsinR* is accompanied by a marked cytokinesis block, as has been seen previously with many gene products involved in trafficking, (Fig. 5C,D). TbCALM-knockdown (KD) cells undergo several rounds of organelle duplication and accumulate more than two nuclei and kinetoplasts (the region of the trypanosome single mitochondrion that contains all mtDNA) – an abnormal state of the cell cycle termed >2N2K (Fig. 5C,D). In contrast, TbEpsinR-KD cells show a block in cytokinesis after one round of mitosis, accumulating mostly two nuclei and two kinetoplasts only (2K2N). Interestingly, silencing of both gene products mimics the effect seen for depletion of TbEpsinR (Fig. 5C), suggesting that the absence of TbEpsinR prevents additional rounds of organelle replication.

### TbCALM and TbEpsinR function synergistically in clathrin-mediated endocytosis

**Fig. 6.**
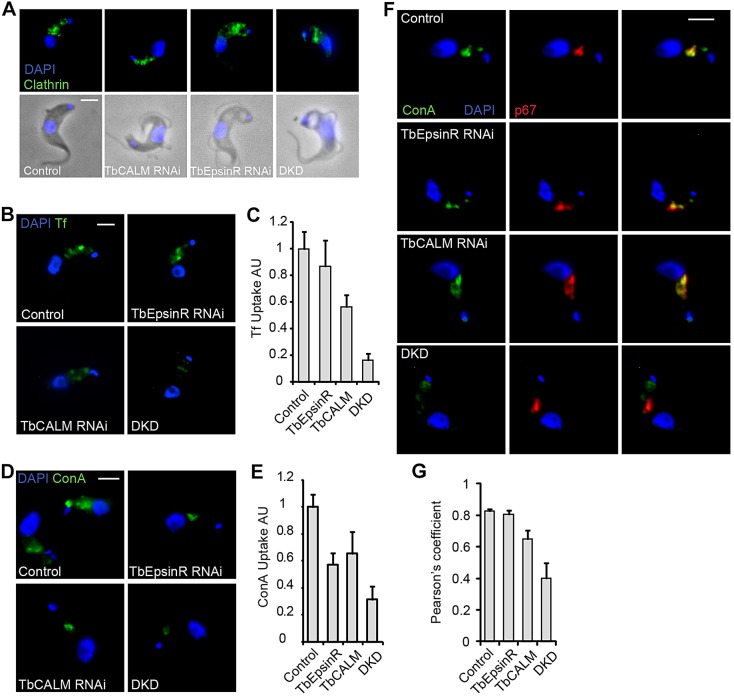
**Endocytic inhibition following TbCALM and TbEpsinR depletion.** (A) Immunofluorescence analysis of clathrin heavy chain distribution (green) in parental (control) and TbCALM, TbEpsinR or double RNAi cell lines (DKD) after 48 h post-induction. Blue indicates DNA DAPI-stain. Note phase-light vacuolar structures reminiscent of the big-eye phenotype observed following clathrin heavy chain depletion (Allen et al., 2003). (B) Uptake of Alexa-Fluor-488-conjugated transferrin in parental (control) and TbCALM, TbEpsinR or double (DKD) RNAi cell lines at 48 h post-induction. (C) Quantification of transferrin as in B. Endocytic inhibition following knockdown of TbEpsinR and TbCALM appears additive, suggestive of some redundancy. Results are mean±s.e.m. (*n*=50). (D) Uptake of FITC-conjugated ConA in parental and TbCALM, TbEpsinR or double (DKD) RNAi cell lines after 48 h post-induction. (E) Quantification of ConA uptake shown in D. Results are mean±s.e.m. (*n*=50). Co-depletion of TbCALM and TbEpsinR leads to greater inhibition of ConA uptake than depletion of either protein alone. (F) Lysosomal (p67, red) delivery of ConA in knockdown cell lines after 48 h RNAi induction. Whereas uptake is reduced (D,E), ConA trafficking to the lysosome appears largely unaffected in either TbCALM or TbEpsinR knockdown cells compared to parental control. However, some distension of the p67-positive compartment is apparent following TbCALM depletion. In contrast, ConA uptake appears largely stalled at the flagellar pocket region in the double knockdown. (G) Quantification of ConA and p67 colocalisation following 48 h RNAi induction. Co-depletion of TbCALM and TbEpsinR greatly reduces lysosomal delivery of ConA. Results are mean±s.e.m. (*n*=50). Scale bars: 2 μm.

Depletion of the clathrin heavy chain in *T. brucei* leads to a complete inhibition of endocytosis accompanied by a dramatic expansion of the flagellar pocket, a useful morphological marker for endocytic dysfunction in *T. brucei* (Allen et al., 2003). Therefore, to assess the effects of TbCALM and TbEpsinR depletion upon CME, we first examined the distribution of clathrin heavy chain and the appearance of enlarged flagellar pockets at 48 h post-RNAi induction. In control cells, clathrin antisera labels tubulovesicular structures arranged between the cell posterior and the nucleus (Fig. 6A), the region containing the entire endosomal system (Morgan et al., 2001; Allen et al., 2003). A subset of clathrin-positive structures are found close to the kinetoplast. This cluster of clathrin punctae represents structures at, or near to, the flagellar pocket, likely newly forming CCPs or early endocytic vesicles still bearing a clathrin coat. TbCALM depletion had little effect on the distribution of clathrin (Fig. 6A), even in severely deformed cells arising from the generalised cytokinesis block (data not shown). Effects of TbEpsinR depletion on clathrin distribution were subtle and in keeping with our previous study (Gabernet-Castello et al., 2009), as it became less clearly tubulovesicular in nature and more diffuse or cytosolic (Fig. 6A). Following depletion of both TbEpsinR and TbCALM together a remarkable enlargement of the flagellar pocket (visible as phase-light vacuoles at the cell posterior) was observed. Enlarged flagellar pockets were seen in over 40% of double KD cells versus 16% and 7% for TbEpsinR and TbCALM single KDs, respectively. Note, however, that these observed proportions are likely an underestimate of the total phenotypic penetrance, as this effect is rapidly lethal, thereby removing severely affected cells from the population.

Flagellar pocket enlargement is a good, although indirect, indicator of endocytic inhibition. To assay this directly, we examined the uptake of two fluorescein-labelled endocytic markers, transferrin and ConA. In trypanosomes, transferrin is taken up by a unique heterodimeric, GPI-anchored transferrin receptor (TbTfR), which has no homology to the mammalian receptor (Steverding, 2000). After a 45-min exposure to transferrin–FITC, control cells showed extensive uptake of the fluorophore, with several punctae visible between the nucleus and the kinteoplast (Fig. 6B,C). Depletion of either TbEpsinR or TbCALM had only a small effect on transferrin uptake, whereas depletion of both together caused a strong reduction. To rule out specific effects upon TbTfR trafficking, we examined the uptake of the mannose-binding lectin ConA. At the trypanosome surface the major mannose-containing molecule is VSG, which owing to its high packing density is likely endocytosed non-concentratively through bulk uptake (Grünfelder et al., 2003). Thus FITC–ConA uptake reports general clathrin-mediated endocytic activity. As seen for transferrin–FITC uptake, depletion of both TbEpsinR and TbCALM together led to a greater inhibition of FITC–ConA uptake than depletion of either protein alone (Fig. 6D,E).

When endocytosis is inhibited in trypanosomes, ConA still accumulates within the flagellar pocket. Following TbCALM and TbEpsinR co-depletion, the ConA–FITC signal was frequently restricted to a single puncta adjacent to the kinetoplast, suggesting that uptake might be stalled at the flagellar pocket. To test this, we examined the delivery of endocytosed ConA–FITC to later endosomal compartments. After depletion of either TbCALM or TbEpsinR alone, endocytosed ConA largely colocalised with p67, indicative of a presence in lysosomes, whereas depletion of both TbCALM and TbEpsinR together greatly reduced the colocalisation of ConA and p67, supporting a block of ConA uptake from the flagellar pocket (Fig. 6F,G). Interestingly, given the role of CALM and AP180 in vacuolar maintenance in *D. discoideum* (Stavrou and O'Halloran, 2006), in many CALM-depleted cells the p67-labelled compartment appeared distended when compared to control cells (Fig. 6F).

### ENTH and ANTH protein depletion affects flagellar pocket function

**Fig. 7.**
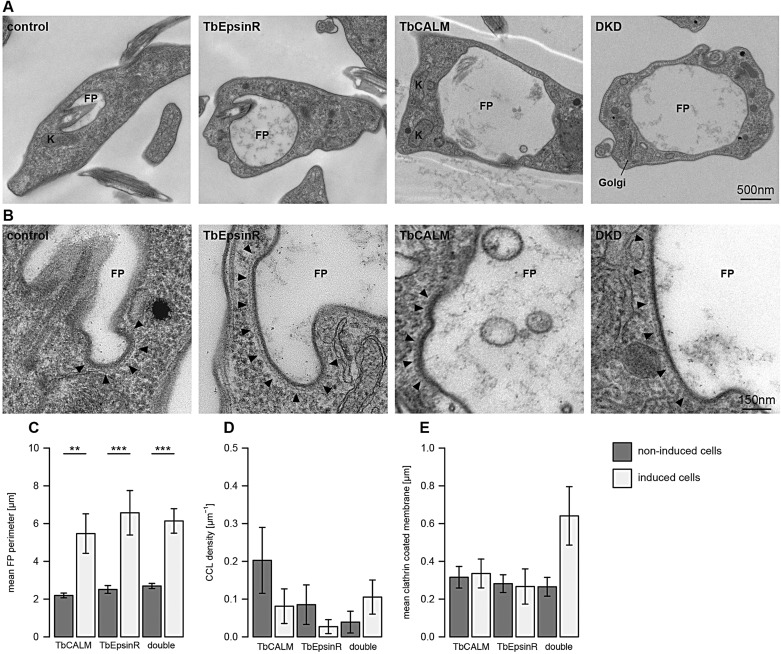
**Defects at the flagellar pocket following ENTH and** **ANTH protein depletion.** (A) Representative electron micrographs showing the great increase in flagellar pocket diameter following depletion of TbEpsinR, TbCALM or both together. (B) Representative micrographs of altered morphology of clathrin-coated structures in RNAi induced cells. Black arrowheads indicate clathrin assembly. (C–E) Bar graphs depict morphometric analysis for each cell line from cultures after 48 h with or without tetracycline. Results are mean±s.e.m. (*n*=25); *P≤0.01, **P≤0.002, ***P≤0.0002.

To further investigate the roles of TbCALM and TbEpsinR in early clathrin recruitment and vesicle formation, we examined RNAi cell lines by fast isothermal fixation and transmission electron microscopy. The most apparent gross abnormality of cells depleted of the ENTH and ANTH proteins was the significant increase in flagellar pocket size (Fig. 7A,C). This is consistent with the compromised uptake of transferrin and ConA (Fig. 6). Interestingly, the endocytic block is not caused by a failure in recruiting clathrin per se: clathrin-coated structures resembling those from control cells are visibly associated with the membrane of enlarged pockets (Fig. 7C–E), either as CCPs or flat lattices (CCLs) (Fig. 7F). There was, however, a reduction in CCP density seen across all RNAi cell lines (Fig. 7D). Close inspection of the morphology of clathrin-coated structures showed aberrant coated pit morphology (Fig. 7B). These defects were less severe and less commonly observed in TbCALM-depleted cells, where pits were still formed, although with irregular morphologies (Fig. 7B), which is qualitatively similar to the defects reported following CALM depletion in mammalian cells (Sahlender et al., 2013). In TbEpsinR-depleted cells, the clathrin-coated membrane regions no longer resembled true pits and instead formed large flat coated areas showing modest curvature at their peripheries (Fig. 7B). This defect was accentuated in tandem knockdown cells, with many instances of extremely large flat clathrin-coated areas (Fig. 7B). Indeed in the tandem knockdown cells, the large accumulation of these aberrant structures was reflected in an overall increase in the amount of coated membrane per section (Fig. 7E).

The Golgi remained largely unaffected, despite the expected importance of clathrin and TbEpsinR to its maintenance (supplementary material Fig. S3). Whereas superficially this result argues against a role for ENTH and ANTH domain proteins at the trypanosome Golgi, it is possible that the extreme dependence of bloodstream-form parasites on clathrin function at the flagellar pocket leads to cell death prior to an observable impact on Golgi morphology. Similarly, ablation of clathrin or clathrin-associated proteins (TbCAPs) in bloodstream-form parasites does not cause any obvious Golgi phenotype, whereas depletion in the less endocytically active insect form is associated with Golgi hypertrophy (Allen et al., 2003; Adung'a et al., 2013).

### TbCALM functions in lysosomal duplication and segregation

**Fig. 8.**
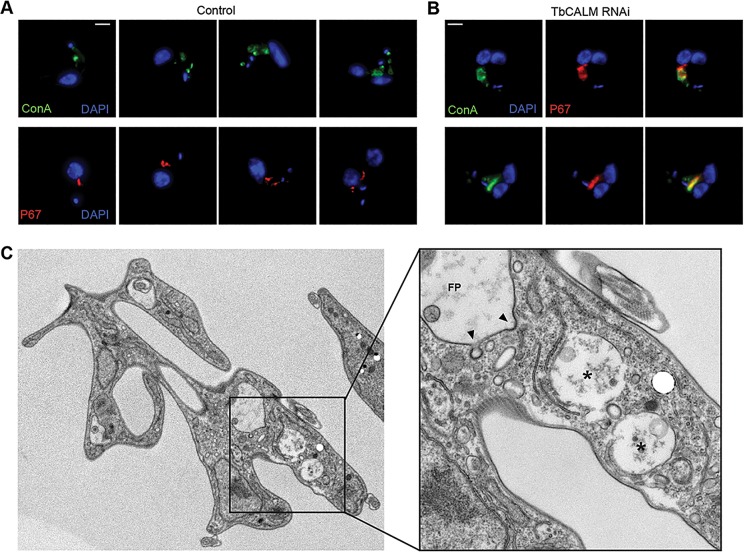
**Lysosomal duplication and segregation defects after TbCALM depletion.** (A) Duplication and segregation of the endosomal system through the cell cycle in parental cells (control). Incubation in the continuous presence of ConA (top panels, green) labels the entire endosomal system. ConA-labelled structures duplicate and segregate in synchrony with kinetoplast duplication until in pre-cytokinesis (i.e. two kinetoplasts and two fully segregated nuclei) two endosomal systems are visible and apparently both competent for endocytosis. p67-positive lysosome (bottom panels, red) apparently undergoes binary duplication and segregation, concurrent with the endocytic system as a whole. (B) TbCALM depletion causes distension of the p67- (red) and ConA- (green) positive compartment, and retardation or block of segregation (two examples are shown). (C) Thin-section transmission electron microscopy of TbCALM-depleted cells (48 h post RNAi induction). The cytokinesis defect is demonstrated by a hugely distorted cell containing two flagellar pockets (FP) and two nuclei. The inset shows a higher magnification of vacuolar structures (indicated by asterisks) suggested to be lysosomal. When present at the thin-section across a flagellar pocket, CCPs show normal morphological appearance (arrowheads).

The distended p67-positive compartment and cytokinesis failure specifically affecting TbCALM-depleted cells led us to ask whether this protein also played a non-redundant role in vacuole maintenance that was distinct from its involvement with CME at the flagellar pocket. To identify the origin of the lysosomal defect, we first characterised the duplication and segregation of this organelle throughout the cell cycle, both alone and in relation to the rest of the endocytic system (Fig. 8). Cells were allowed to continually accumulate FITC–ConA for 45 min prior to fixation, and then examined for cell cycle positioning by DAPI staining of kinetoplast and nuclear DNA. Endosomal organelle duplication appears to initiate from the flagellar pocket (Fig. 8A, top panels), consistent with the role of the flagellum, basal body and kinetoplast in the timing of cell division. In the time between kinetoplast duplication and mitosis, the rest of the endosomal system is duplicated, leading to two complete and patent pathways prior to cytokinesis. Lysosomal duplication apparently occurs by binary fission soon after kinetoplast segregation (Fig. 8A, lower panels), in line with an earlier report linking lysosomal and mitochondrial duplication and segregation in trypanosomes (Vanhollebeke et al., 2010). In TbCALM-depleted cells, there were clear defects to lysosomal segregation (Fig. 8B) leading to large, apparently continuous structures forming in post-mitotic cells. This observation was confirmed at the ultrastructural level (Fig. 8C) where often there were multiple structures located in close apposition, again suggesting errors to lysosomal segregation.

## DISCUSSION

Extending our knowledge of conserved and novel cellular processes across eukaryotic lineages is essential in providing new perspectives and insights into how evolution has remodelled familiar pathways, adapting them to match specific selective pressures, and which might be relevant to disease-linked processes. Such studies can also identify conserved core features, providing insights into fundamental aspects of cell biology and the configuration of ancestral states. *T. brucei* provides an excellent example of this evolutionary adaptation, where the selective pressure for survival within the host bloodstream is counteracted by extremely rapid endocytic flux, rapidly clearing the parasite surface of host antibodies (Engstler et al., 2007; Field and Carrington, 2009; Manna et al., 2014). This fast endocytic activity is essential and solely dependent upon clathrin (Allen et al., 2003). CME was a feature of the last eukaryotic common ancestor and is correspondingly found across all lineages. Historically, there has been debate over the relative contribution of CME to total endocytic activity in mammalian cells but a recent analysis suggests that nearly all endocytic activity also relies upon clathrin in unperturbed mammalian cells (Bitsikas et al., 2014). Comparative genomics demonstrates an ancient and conserved core of widely distributed genes together with animal and fungal specific innovations, largely comprising cargo-specific adaptors (Field et al., 2007; Koumandou et al., 2013). Surprisingly, given their dependence upon CME for survival, *T. brucei* lacks a central conserved component, the heterotetrameric AP2 complex (Field et al., 2007; Manna et al., 2013), although it retains the clathrin-associated adaptor proteins TbCALM and TbEpsinR.

TbCALM and TbEpsinR represent members of two well-conserved families with wide eukaryotic distributions. Both have conserved domain architecture with their opisthokont orthologues and sequence conservation within their respective ANTH and ENTH domains, suggestive of conserved functions in phosphoinositide binding. PtdIns(4,5)*P*_2_ plays an important role in endocytosis and flagellar pocket homeostasis in *T. brucei*, although the factors required to recognise this lipid are unknown (Demmel et al., 2014). Consistent with PtdIns(4,5)*P*_2_ at the flagellar pocket, we demonstrate phosphoinositide-dependent plasma membrane targeting for both TbEpsinR and TbCALM when expressed in mammalian cells. Whereas yeast and mammalian EpsinRs preferentially bind to PtdIns(4)*P* and show strong Golgi localization, we find that TbEpsinR has at least some binding affinity for plasma membrane PtdIns(4,5)*P*_2_ when expressed in COS-7 cells. Close inspection of the conservation of residues involved in phosphoinositide binding in the Epsins and EpsinR lends support to an intermediate phosphoinositide selectivity of TbEpsinR (Gabernet-Castello et al., 2009). However a more detailed functional analysis is required before firm conclusions can be drawn with respect to the precise lipid specificities of TbCALM and TbEpsinR *in vivo*. Nevertheless, we suggest that, in the absence of the AP2 complex or other PtdIns(4,5)*P*_2_-sensing CCP nucleators such as EPS15, EPS15R or muniscins (Naslavsky et al., 2007; Stimpson et al., 2009), TbCALM and TbEpsinR are able to function as an important link between flagellar pocket phosphoinositides [likely PtdIns(4,5)*P*_2_] and CCP formation.

Selection of cargoes for incorporation into CCPs is usually mediated by an interaction of short linear motifs within the intracellular domains of proteins with specific cargo or clathrin adaptors. These motif–adaptor interactions are well studied and include the interaction of AP complexes with Yxxɸ and [D/E]xxxL[LI] motifs as well as GGA protein family interactions with DxxLL and ARH binding to FxNPxY motifs (reviewed in Kelly and Owen, 2011). In contrast, both EpsinR and CALM interact with specific soluble NSF attachment protein receptors (SNAREs) through relatively complex and specific folded domain interactions (Miller et al., 2007; Miller et al., 2011).

So how then might this system function in *T. brucei*? Firstly, we can disregard cargo adaptors of the GGA and phosphotyrosine binding (PTB) domain types, such as ARH and DAB, as these arose well after divergence of the excavate lineage and are absent from the genomes of non-opisthokont organisms (Field et al., 2007). This leaves the ancient adaptors AP2, CALM and EpsinR. Although both EpsinR and CALM are far more cargo selective than AP2, there is no evidence that cargo binding and clathrin recruitment are linked. In fact, two CALM mutants deficient for SNARE binding are still able to drive correct CCP formation (Sahlender et al., 2013). In this way, it is possible that *T. brucei* has adopted a predominantly cargo-independent means of CCP formation while retaining the important downstream ability to selectively sort specific SNAREs to their correct locations. This model suggests an uncoupling of CCP formation from cargo binding at the plasma membrane, which we believe might underlie the fast kinetics of CME in *T. brucei*.

Importantly, loss of AP2 from the African trypanosome lineage was concurrent with emergence of the GPI-anchored, and thus non-AP2 interacting, VSG coat, at least from the resolution available from sequenced genomes (Field et al., 2007; Manna et al., 2013). Given that the high rate of endocytosis in *T. brucei* underlies the removal of host-antibody-bound VSG from the surface, any concentration of non-VSG, AP2-interacting cargoes into nascent endocytic pits would likely result in decreased efficiency of surface VSG removal. Additionally, the interaction of AP2 complexes with the membrane is further stabilised by cargo binding (Jackson et al., 2010). Thus, the efficiency of AP2-driven CCP formation would be predicted to be inversely related to the relative concentration of VSG versus non-VSG cargoes. This suggests that abandoning the cargo- and AP2-dependent mechanism of CCP formation would aid in packaging more VSG into each endocytic vesicle. Furthermore, the absence of AP2 might be fundamentally related to the extreme rapidity of CME in trypanosomes as AP2 appears not to be required in synaptic vesicle endocytosis, another example of a highly specialized and rapid endocytosis (Willox and Royle, 2012). The similarities between these two systems are a potential example of convergent evolution, and indicate that AP2 probably provides a selective disadvantage in circumstances where speed is paramount. We are not, however, intending to draw other mechanistic parallels between neuronal ‘fast CME’ and *T. brucei* CME, as there is no evidence to support multiple distinct modes of CME in *T. brucei*. Although necessarily highly speculative, these two lines of reasoning lead us to suggest that the combination of superabundant GPI-anchored surface cargo and pressure for rapidity might underlie the unique configuration of the CME pathway in *T. brucei.*

In addition to its role in endocytic CCP formation, TbCALM appears to be important for proper duplication and segregation of the lysosome in *T. brucei*. As stated above, the SNARE trafficking function of human CALM can be perturbed independently from its endocytic role (Sahlender et al., 2013). It is possible therefore that the endocytic effects of TbCALM depletion are masked by TbEpsinR functioning redundantly at the flagellar pocket, whereas a specific and non-redundant SNARE sorting defect gives rise to the observed lysosomal phenotype. In *Dictyostelium*, CALM knockout leads to deregulation of contractile vacuole size through VAMP7B mis-sorting, resulting in endosomal fusion defects (Stavrou and O'Halloran, 2006; Wen et al., 2009). A similar mechanism would seem to explain the observed lysosomal phenotype in TbCALM-depleted *T. brucei.* However, a direct assessment of the effects of TbCALM depletion on SNARE sorting is needed to confirm this hypothesis, and presently there is little understanding of trypanosome SNARE function (Murungi et al., 2014).

In summary, the current study details partially redundant functions for ENTH and ANTH domain protein homologues in CCP formation in *T. brucei*, allowing for rapid and AP2-independent CME. We have also confirmed an important and evolutionarily conserved role for membrane phosphoinositides and their adaptors in CME, extending beyond the requirement for AP2 recruitment.

## MATERIALS AND METHODS

### Cell culture and transfection

Bloodstream-form *Trypanosoma brucei* strain Lister 427 parasites were cultured in HMI-9 medium supplemented with 10% fetal bovine serum (Hirumi and Hirumi, 1989). For RNAi experiments, the tetracycline-responsive single-marker bloodstream-form cell line was maintained under G418 selection (Wirtz et al., 1999). For trypanosome transfections 3×10^7^–4×10^7^ cells in mid-log phase (1×10^6^ cells/ml) were transfected with 10 µg of DNA using an AMAXA nucleofection system and the human T-cell nucleofection kit (Lonza). Stably transformed clonal cell lines were then selected by limiting dilution in the presence of appropriate antibiotics. Antibiotics used were G418 (2 µg/ml), hygromycin B (5 µg/ml), puromycin (0.2 µg/ml). COS-7 cells were cultured in Dulbecco's modified Eagle's medium (DMEM) supplemented with 10% fetal bovine serum and transiently transfected with Fugene HD reagent.

### RNA interference

For TbCALM and TbEpsinR single knockdowns, suitable silencing fragments were identified by RNAit software (Redmond et al., 2003) and amplified by PCR from genomic DNA using Taq DNA polymerase. Primers for TbEpsinR RNAi were: forward, 5ʹ-TTGTCGTGTCTTCCAAGCTG-3ʹ and reverse, 5ʹ-CATACGCTGTGCCTCAGAAA-3ʹ, giving a 556-bp fragment. Primers for TbCALM were forward, 5ʹ-TCTTTGAGTCGCTGTTGGTG-3ʹ and reverse, 5ʹ-TGAAGTTGTCGCCTTCAGTG-3ʹ, giving a 446-bp fragment. These gene fragments were cloned into the p2T7^Ti^:TAblue vector between opposing tetracycline-inducible T7 promoters to drive dsRNA expression (Alibu et al., 2005) in single-marker bloodstream-form cells. For the TbEpsinR and TbCALM double knockdown, the TbEpsinR fragment was amplified using the modified forward primer 5ʹ-GACCTAGCGTCTTGTCGTGTCTTCCAAGCTG-3ʹ containing an Eam1105i site. After cloning into p2T7^Ti^:TAblue, the construct was digested with Eam1105i, blunted with T4 DNA polymerase and T overhangs were added with Taq in the presence of dTTP alone. The TbCALM RNAi fragment was then cloned into this vector as above, generating a single construct expressing a dsRNA fragment targeting both TbEpsinR and TbCALM. For all cell lines, multiple clonal populations were obtained under hygromycin B and G418 selection. Following RNAi induction with tetracycline (1 µg/ml), knockdown efficiency was assessed by qRT-PCR.

### *In situ* tagging of TbCALM.

GFP or 3×HA tags were introduced to the C-terminus of TbCALM by homologous recombination of a tagging cassette into the genomic locus using the pMOTag system (Oberholzer et al., 2006). Forward tagging primer, 5ʹ-CGTCAGCATCATGGGGTCGAGGTAATTGCGGTAGCAA-TACTGTGGATCCGTTTAAGGATCTTTACGCGAGCCAGAAGGGA-GGCCAGGGTACCGGGCCCCCCCTCGAG-3ʹ * *and reverse tagging primer, 5ʹ-TAAGGACACAGTATTTTACCCAGACCCAACCACTGCACCAAC-ACACGACCTGAATAATTGGAAAACGTTTTCATCCTGCCACTCGA-TGGCGGCCGCTCTAGAACTAGTGGAT-3ʹ were used to amplify a tagging cassette from either pMOT3G, bearing a GFP tag and G418 selectable marker, or pMOT23H, bearing a 3×HA tag and puromycin selectable marker. PCR purification and transfection were performed as previously described (Oberholzer et al., 2006). Correct integration of the tagging cassette was assessed by western blot.

### Immunofluorescence of trypanosomes

Mid-log phase parasites harvested by centrifugation at 800 ***g***, 4^°^C for 10 min were washed once in chilled, serum-free HMI-9 and fixed in ice-cold 2% paraformaldehyde in phosphate-buffered saline (PBS). Cells were then adhered to poly-L-lysine-coated slides and fixative removed by two washes in PBS. Adhered cells were permeabilised with 0.2% Triton X-100 in PBS for 10 min and blocked in 20% fetal bovine serum plus 0.1% Triton X-100 in PBS for 1 h. All antibody incubations were carried out for 1 h at room temperature in blocking solution. Polyclonal rabbit anti-clathrin heavy chain antibody (Morgan et al., 2001) was used at 1:2500. Polyclonal rabbit anti-GFP antibody, a kind gift from Mike P. Rout (Rockefeller University, NY, USA) was used at 1:2000. Mouse monoclonal anti-P67 was a kind gift from James D. Bangs (University at Buffalo, SUNY, NY, USA) and used at 1:2000. Rat monoclonal anti-HA antibody (clone 3F10, Roche) was used at 1:1000. Mouse monoclonal anti-clathrin X22 antibody was a kind gift from Margaret Robinson (Cambridge Institute for Medical Research, Cambridge, UK). Appropriate Alexa-Fluor-conjugated secondary antibodies (Life Technologies) were used at 1:2000.

### PtdIns(4,5)*P*_2_ depletion assay

For expression in COS-7 cells, the entire TbEpsinR open reading frame (ORF) was amplified from genomic DNA using forward primer 5ʹ-GTACGAGATCTATGTCATTTCCGACTTCTCTCC-3ʹ * *and reverse primer 5ʹ-GTACGGAATTCCTGACCTAACCGGCGACC-3ʹ, and cloned into pEGFP-N2 between the BglII and EcoRI sites. TbCALM was amplified with forward primer 5ʹ-ACTTGGTCGACGGATGAACTCTAAAGACACGAATGAGTTG-3ʹ and reverse primer 5ʹ-GCTATCCGCGGATCTGGCCTCCCTTCTGGCT-3ʹ, and cloned into pEGFP-N2 between the SalI and SacII sites. GFP–C1-PLCδ-PH, Lyn11-targeted FRB and CFP–Inp54p were from Addgene (Cambridge, MA, USA; plasmids numbers 21179, 20147 and 20155, respectively; Suh et al., 2006). COS-7 cells were seeded onto glass coverslips and allowed to adhere overnight prior to transfection with either TbEpsinR–EGFP, TbCALM–EGFP or GFP–PLCδ-PH together with Lyn11–FRB and CFP–Inp54p. At 48 h post-transfection cells were rinsed with PBS and incubated in serum-free DMEM with or without rapamycin (10 µM) for 30 min at 37°C. Cells were then rinsed with ice-cold PBS and fixed in 2% paraformaldehyde in PBS on ice for 10 min. Coverslips were mounted onto slides in Prolong Gold (Life Technologies) for imaging.

### Endocytosis assays

Endocytic uptake was assayed as previously described (Gabernet-Castello et al., 2009). Briefly, cells were washed in chilled, serum-free HMI-9 and resuspended at 10^7^ cells/ml in chilled, serum-free HMI-9 plus 1% BSA containing either fluorescein-conjugated concanavalin A (5 μg/ml) or Alexa-Fluor-488-conjugated human transferrin (25 μg/ml). Cells were then either kept on ice (0-min timepoint) or transferred to 37°C for the desired time before being washed three times in chilled PBS. Following the pulse, cells were fixed in ice-cold 2% paraformaldehyde in PBS and processed for immunofluorescence as above. Samples were imaged under identical acquisition settings and dye uptake was quantified using the publicly available ImageJ software (National Institutes of Health, rsbweb.nih.gov/ij).

### Fast, isothermal fixation and electron microscopy

To minimise perturbations to endocytosis due to live cell handling, the cell lines analysed ultrastructurally were grown to mid-log phase and rapidly fixed in culture by the addition of isothermal glutaraldehyde to the culture flask, to a final concentration of 2.5%, as previously described (Gadelha et al., 2009). The culture flask was gently rocked for 10 min at 37°C, after which time fixed cells in medium were harvested by centrifugation at 800 ***g*** for 10 min and resuspended in 2.5% glutaraldehyde in PBS for another 30 min at room temperature. Fixed cells were post-fixed in 1% osmium tetroxide in PBS for 30 min at room temperature, en-bloc-stained with 1% aqueous uranyl acetate, dehydrated through acetone and embedded in epoxy resin. Ultra-thin sections (70 nm) were post-stained with 2% aqueous uranyl acetate and lead citrate. For morphometric analysis, measurements on electron micrographs were done using ImageJ. Bar graphs and tests in Fig. 7 were performed using the statistical programming package ‘R’ (The R Project for Statistical Computing, r-project.org).

### qRT-PCR

RNA was isolated with an RNeasy Mini Kit (Qiagen, Manchester, UK) according to the manufacturer's instructions. First strand cDNA was synthesised from 1 μg total RNA using Superscript III reverse transcriptase (Invitrogen) with oligo(dT) primer. For qRT-PCR, the cDNA template was amplified using a iQ-SYBRGreen supermix (Bio-Rad) and a Mini-Opticon Real-Time PCR system (Bio-Rad). Expression levels were normalised to those of β-tubulin. Primers used were TbEpsinRqRTF, 5ʹ-CTCAATCACCACCTTTGTCG1-3ʹ; TbEpsinRqRTR, 5ʹ-TGTGCGATTTGTTGTTCCAT; TbCALMqRTF, 5ʹ-TCAAAACTTCTTCGGCCAAC-3ʹ; TbCALMqRTR, 5ʹCCATGATGCTGACGAATCAC-3ʹ; TbbTubqRTF, 5ʹ-CAAGATGGCTGTCACCTTCA-3ʹ; and TbbTubqRTR, 5ʹ-GCCAGTGTACCA-GTGCAAGA.

### Comparative genomics and phylogenetics

Homology searches were performed with BLAST and human and yeast genome sequences as queries; retrieved sequences returning an e-value below 1×10^−3^ were verified by reciprocal BLAST against the human or yeast databases as appropriate. Genome databases searched were: *Homo sapiens*, *Saccharomyces cerevisiae*, *Batrachochytrium dendrobatidis*, *Entamoeba histolytica*, *Dictyostelium discoideum*, *Arabidopsis thaliana*, *Physcomitrella patens*, *Micromonas pusilla*, *Cyanidioschizon merolae*, *Paramecium tetraurelia*, *Thalassiosira pseudonana*, *Phytophthora ramorum* (NCBI, www.ncbi.nlm.nih.gov); *Salpingoeca rosetta* (Origins of Multicellularity database, Broad Institute, www.broadinstitute.org); *Paramecium falciparum* (PlasmoDB, www.plasmodb.org); *Toxoplasma gondii* (ToxoDB, www.toxodb.org); *Naegleria gruberi* (Joint Genome Initiative, US Department of Energy, http://genome.jgi.doe.gov); *Bodo saltans* (GeneDB, www.genedb.org); *Leishmania major*, *Trypanosoma cruzi*, *Trypanosoma brucei* (TriTrypDB, www.tritrypdb.org); and *Giardia lamblia* (GiardiaDB, www.giardiadb.org). Where no orthologues were identified, further searches were carried out using relevant sequences from closely related taxa where available. Retrieved sequences were parsed through the NCBI conserved domain database (CDD) and HMMScan (HMMER, Janelia, http://hmmer.janelia.org) to identify conserved domains. A candidate sequence was considered orthologous based upon the presence of conserved domains, high scoring (e-value below 1×10^−3^) reciprocal BLAST returning the initial query sequence amongst the top hits, and inspection of protein sequence alignments (MAFFT) for conserved regions. Where these tests were not met, the query gene or gene family was considered not found within the target taxon. For phylogenetic reconstruction, protein sequences were aligned using MergeAlign (www.mergealign.appspot.com; Collingridge and Kelly, 2012) and edited manually to remove gaps and poorly conserved regions. Phylogenetic trees were reconstructed using Bayesian (MrBayes) and maximum likelihood (RaxML, PhyML) approaches. PhyML was run through the South of France Bioinformatics Platform web server (www.atgc-montpellier.fr/phyml). RaxML and MrBayes were run through the Cyberinfrastructure for Phylogenetic Research (CIPRES) Science Gateway web server (www.phylo.org). MrBayes version 3.1.2, analyses were run using a mixed model for 1×10^6^ generations, with convergence verified by standard deviation of split frequencies <0.05. All tress before plateau were removed as burn-in. For ML approaches the appropriate model was assessed by protest v2.4 (http://darwin.uvigo.es/software/prottest2_server.html).
